# Bis(μ-3,5-dinitro­benzoato-κ^2^ *O* ^1^:*O* ^1′^)bis­(μ-3,5-dinitro­benzoato)-κ^3^ *O* ^1^,*O* ^1′^:*O* ^1^;κ^3^ *O* ^1^:*O* ^1^,*O* ^1′^-bis­[(3,5-dinitro­benzoato-κ^2^ *O* ^1^,*O* ^1′^)(1,10-phenanthroline-κ^2^ *N*,*N*)dysprosium(III)]

**DOI:** 10.1107/S1600536810052001

**Published:** 2010-12-18

**Authors:** Chun-Hua Dong, Da-Hai Zhang, Ning Ren, Gai-Qing Xi, Juan-Juan Hao

**Affiliations:** a Department of Chemistry, Handan College, Handan, Hebei 056005, People’s Republic of China

## Abstract

In the binuclear title complex, [Dy_2_(C_7_H_3_N_2_O_6_)_6_(C_12_H_8_N_2_)_2_], the Dy^III^ ions exhibit a distorted monocapped square-anti­prismatic geometry and are coordinated by seven O atoms of four 3,5-dinitrobenzoate (DNBA) anions and two N atoms of a phenanthroline ligand. The carboxylate groups of the DNBA anions exhibit three coordination modes: bidentate chelating, bidentate chelating–bridging and tridentate chelating–bridging. The center of the mol­ecule is located on a crystallographic center of inversion.

## Related literature

For related structures, see: Wang *et al.* (2004 ▶); Ren *et al.* (2006 ▶); Zhang *et al.* (2007 ▶); Xu *et al.* (2008*a* ▶,*b* ▶). 
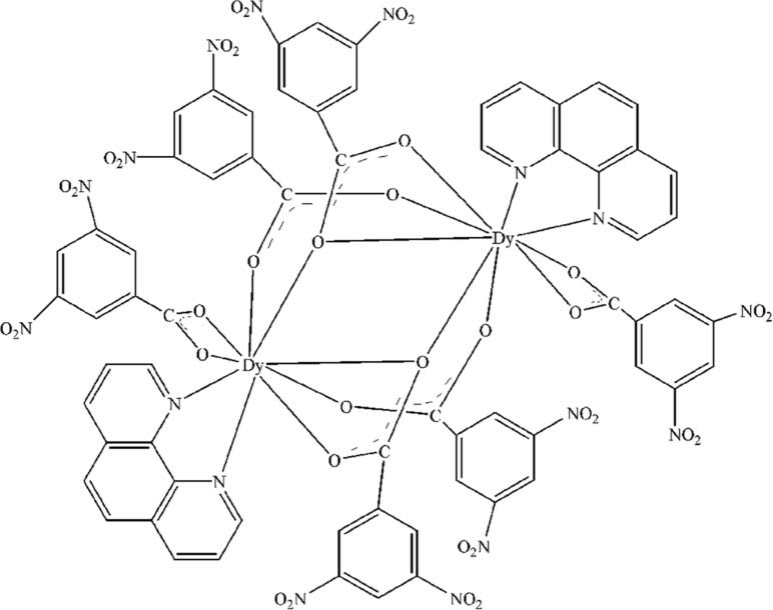


## Experimental

### 

#### Crystal data

[Dy_2_(C_7_H_3_N_2_O_6_)_6_(C_12_H_8_N_2_)_2_]
*M* *_r_* = 1952.09
Triclinic, 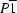

*a* = 11.9569 (4) Å
*b* = 12.8636 (4) Å
*c* = 13.1187 (4) Å
α = 104.24 (5)°
β = 113.96 (5)°
γ = 100.46 (5)°
*V* = 1694.6 (12) Å^3^
*Z* = 1
Mo *K*α radiation
μ = 2.31 mm^−1^
*T* = 296 K
0.15 × 0.13 × 0.12 mm

#### Data collection

Bruker APEXII CCD diffractometer
Absorption correction: multi-scan (*SADABS*; Sheldrick, 1997) ▶ *T*_min_ = 0.724, *T*_max_ = 0.769
8296 measured reflections
5771 independent reflections
5624 reflections with *I* > 2σ(*I*)
*R*_int_ = 0.011

#### Refinement

*R*[*F* ^2^ > 2σ(*F* ^2^)] = 0.016
*wR*(*F* ^2^) = 0.041
*S* = 1.03
5771 reflections
541 parameters
H-atom parameters constrained
Δρ_max_ = 0.43 e Å^−3^
Δρ_min_ = −0.78 e Å^−3^

### 

Data collection: *APEX2* (Bruker, 2004 ▶); cell refinement: *SAINT* (Bruker, 2001 ▶); data reduction: *SAINT*; program(s) used to solve structure: *SHELXS97* (Sheldrick, 2008 ▶); program(s) used to refine structure: *SHELXL97* (Sheldrick, 2008 ▶); molecular graphics: *SHELXTL* (Sheldrick, 2008 ▶); software used to prepare material for publication: *SHELXTL* and local programs.

## Supplementary Material

Crystal structure: contains datablocks I, global. DOI: 10.1107/S1600536810052001/im2251sup1.cif

Structure factors: contains datablocks I. DOI: 10.1107/S1600536810052001/im2251Isup2.hkl

Additional supplementary materials:  crystallographic information; 3D view; checkCIF report

## Figures and Tables

**Fig. 1.**
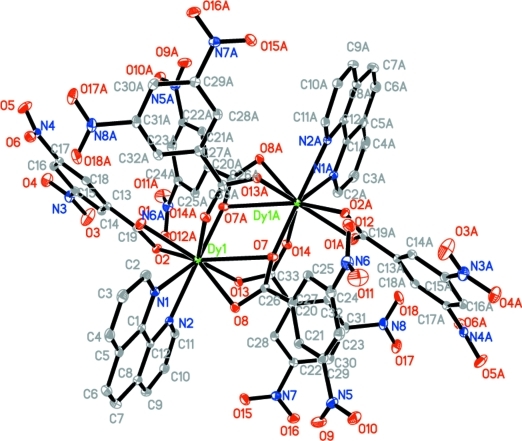
Molecular structure of the title compound with the atom-labelling scheme.

**Fig. 2.**
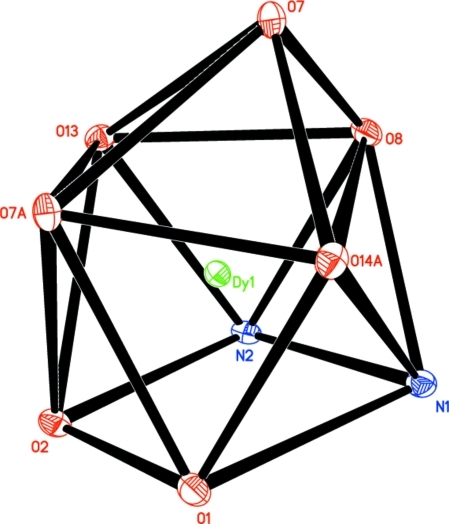
Coordination environment of DyIII ions.

## supplementary crystallographic information

### Comment

Coordination compounds of rare earth metals with various carboxylic acids are of high interest because of their special structures and fascinating properties. Nowadays, they have wide applications to many fields as e.g. new materials (Wang, *et al.*, 2004). Therefore, benzoic acid derivatives and 1,10-phenanthroline were chosen to prepare complexes with a mixed ligand set. As an extention of our previous studies (Ren, *et al.*, 2006; Zhang, *et al.*, 2007; Xu, *et al.*, 2008*a*,*b*), we now report the synthesis and molecular structure of the title dysprosium complex with 3,5-dinitrobenzoic acid and 1,10-phenanthroline.

The binuclear molecular structure of [Dy(3,5-DNBA)_3_(phen)]_2_ is shown in Fig. 1. Fig.2 shows the coordination geometry about the Dy^III^ ions. Two Dy^III^ ions are linked by two bidentate chelating-bridging and tridentate chelating-bridging carboxylate groups. Each Dy^III^ ion is ninefold coordinated to two nitrogen atoms (N1, N2) from one 1,10-phenanthroline molecule, two oxygen atoms (O1, O2) from one bidentate chelating carboxylate group, two oxygen atoms (O13, O14A) from bidentate chelating-bridging carboxylate groups and three oxygen atoms (O7, O7A, O8) from tridentate chelating-bridging carboxylate groups. The coordination polyhedron adopts a distorted mono-capped square antiprismatic geometry. The oxygen atom (O7) from the tridentate chelating-bridging carboxylate adopts the capped position. The coordination mode is similar to that of [Dy(*p*-MOBA)_3_phen]_2_ (Zhang, *et al.*, 2007) but different from that of [Dy(BA)_3_phen]_2_ (Xu, *et al.*, 2008*a*) and [Dy(*m*-MBA)_3_phen]_2_.H_2_O (Xu, *et al.*, 2008*b*).

In the coordination polyhedron of Dy^III^ ion, Dy—O distances are in the range of 2.3257 (13) to 2.7576 (14) Å, and the mean bond length of Dy—O is 2.4341 Å. The average Dy—N distance is 2.5242 Å. It can be easily seen that the bond of Dy—O is stronger than that of Dy—N in the complex corresponding to HSAB concept. Since carboxylate units are negatively charged the corresponding oxygen atoms are the by far harder ligands and therefore establish stronger bonds towards the hard Lewis acid Dy(III) as compared to neutral nitrogen donor atoms. At the same time, the average Dy—O distance of the title complex (2.4341 Å) is slightly longer than the corresponding average distances of the complexes [Dy(BA)_3_phen]_2_ (Xu, *et al.*, 2008*a*) (2.364 Å); and [Dy(*m*-MBA)_3_phen]_2_.H_2_O (Xu, *et al.*, 2008*b*) (2.346 Å). This effect can be explained by electronic effects of different substituents at the benzoate ligands.

### Experimental

DyCl_3_.6H_2_O was obtained by a reaction of Dy_2_O_3_ (99.95%) and HCl (6.0 mol.*L*^-1^) followed by water bath evaporation. DyCl_3_.6H_2_O (0.06 mmol), 3,5-dinitrobenzoic acid (0.18 mmol), 1,10-phenanthroline(0.06 mmol) and water (1 ml) were mixed in a Parr Teflon-lined stainless vessel (25 ml). After the solution was heated at 150°C for 5 d and cooled to room temperature, yellow block crystals of the title complex were obtained in 30% yield.

### Refinement

H atoms were positioned geometrically and refined using a riding model, with C—H = 0.93 Å and with *U*_iso_(H) = 1.2 times *U*_eq_(C).

### Crystal data

**Table:**

[Dy_2_(C_7_H_3_N_2_O_6_)_6_(C_12_H_8_N_2_)_2_]	*Z* = 1
*M**_r_* = 1952.09	*F*(000) = 962
Triclinic, *P*1	*D*_x_ = 1.913 Mg m^−^^3^
Hall symbol: -P 1	Mo *K*α radiation, λ = 0.71073 Å
*a* = 11.9569 (4) Å	Cell parameters from 9404 reflections
*b* = 12.8636 (4) Å	θ = 2.9–31.0°
*c* = 13.1187 (4) Å	µ = 2.31 mm^−^^1^
α = 104.24 (5)°	*T* = 296 K
β = 113.96 (5)°	Block, yellow
γ = 100.46 (5)°	0.15 × 0.13 × 0.12 mm
*V* = 1694.6 (12) Å^3^	

### Data collection

**Table:**

Bruker APEXII CCD diffractometer	5771 independent reflections
Radiation source: fine-focus sealed tube	5624 reflections with *I* > 2σ(*I*)
graphite	*R*_int_ = 0.011
φ and ω scans	θ_max_ = 25.0°, θ_min_ = 2.9°
Absorption correction: multi-scan (*SADABS*; Sheldrick, 1997)	*h* = −14→14
*T*_min_ = 0.724, *T*_max_ = 0.769	*k* = −14→15
8296 measured reflections	*l* = −15→9

### Refinement

**Table:**

Refinement on *F*^2^	Primary atom site location: structure-invariant direct methods
Least-squares matrix: full	Secondary atom site location: difference Fourier map
*R*[*F*^2^ > 2σ(*F*^2^)] = 0.016	Hydrogen site location: inferred from neighbouring sites
*wR*(*F*^2^) = 0.041	H-atom parameters constrained
*S* = 1.03	*w* = 1/[σ^2^(*F*_o_^2^) + (0.0218*P*)^2^ + 1.1919*P*] where *P* = (*F*_o_^2^ + 2*F*_c_^2^)/3
5771 reflections	(Δ/σ)_max_ = 0.002
541 parameters	Δρ_max_ = 0.43 e Å^−^^3^
0 restraints	Δρ_min_ = −0.78 e Å^−^^3^

### Special details

**Table:**

Geometry. All e.s.d.s (except the e.s.d in the dihedral angle between two l.s. planes) are estimated using the full covariance matrix. The cell e.s.d.s are taken into account individually in the estimation of e.s.d.s in distances, angles and torsion angles; correlations between e.s.d.s in cell parameters are only used when they are defined by crystal symmetry. An approximate (isotropic) treatment of cell e.s.d.s is used for estimating e.s.d.s involving l.s. planes.
Refinement. Refinement of *F*^2^ against ALL reflections. The weighted *R*-factor *wR* and goodness of fit *S* are based on *F*^2^, conventional *R*-factors *R* are based on *F*, with *F* set to zero for negative *F*^2^. The threshold expression of *F*^2^ > σ(*F*^2^) is used only for calculating *R*-factors(gt) *etc*. and is not relevant to the choice of reflections for refinement. *R*-factors based on *F*^2^ are statistically about twice as large as those based on *F*, and *R*- factors based on ALL data will be even larger.

### Fractional atomic coordinates and isotropic or equivalent isotropic displacement parameters (Å^2^)

**Table:**

	*x*	*y*	*z*	*U*_iso_*/*U*_eq_	
Dy1	0.871683 (8)	−0.052102 (7)	0.823196 (7)	0.01272 (4)	
O1	0.74648 (14)	−0.24750 (12)	0.72351 (13)	0.0227 (3)	
O2	0.64232 (13)	−0.13350 (11)	0.76732 (12)	0.0191 (3)	
O3	0.19645 (18)	−0.30876 (16)	0.6887 (2)	0.0513 (5)	
O4	0.11452 (15)	−0.49005 (15)	0.62444 (16)	0.0358 (4)	
O5	0.39257 (16)	−0.72017 (13)	0.60925 (18)	0.0385 (4)	
O6	0.52990 (16)	−0.65952 (13)	0.55170 (14)	0.0284 (3)	
O7	1.11237 (13)	0.08515 (12)	1.00543 (12)	0.0187 (3)	
O8	1.03333 (13)	0.09536 (12)	0.82564 (12)	0.0204 (3)	
O9	1.31759 (17)	0.43985 (14)	0.83671 (16)	0.0362 (4)	
O10	1.51618 (16)	0.44826 (14)	0.88585 (15)	0.0348 (4)	
O11	1.67069 (17)	0.15932 (18)	1.0302 (2)	0.0497 (5)	
O12	1.57894 (15)	0.08509 (13)	1.11887 (14)	0.0274 (3)	
O13	0.85945 (13)	0.11215 (11)	0.93722 (12)	0.0177 (3)	
O14	0.97802 (13)	0.14829 (12)	1.13307 (12)	0.0189 (3)	
O15	0.71846 (17)	0.41054 (14)	0.80972 (14)	0.0334 (4)	
O16	0.79596 (16)	0.58264 (13)	0.93352 (15)	0.0301 (4)	
O17	1.09372 (17)	0.66977 (14)	1.34595 (15)	0.0354 (4)	
O18	1.17536 (17)	0.54349 (15)	1.39998 (14)	0.0380 (4)	
N1	0.88628 (16)	−0.11343 (14)	0.62924 (15)	0.0186 (4)	
N2	0.74346 (15)	0.01781 (14)	0.66740 (14)	0.0176 (3)	
N3	0.20251 (18)	−0.40429 (16)	0.65720 (17)	0.0270 (4)	
N4	0.45681 (17)	−0.64570 (15)	0.59303 (16)	0.0238 (4)	
N5	1.41001 (19)	0.40570 (16)	0.87562 (17)	0.0274 (4)	
N6	1.58388 (17)	0.14053 (16)	1.05651 (17)	0.0267 (4)	
N7	0.78537 (17)	0.48234 (15)	0.90981 (16)	0.0220 (4)	
N8	1.10119 (18)	0.57440 (16)	1.32688 (16)	0.0257 (4)	
C1	0.83068 (19)	−0.06731 (16)	0.54593 (18)	0.0194 (4)	
C2	0.9514 (2)	−0.18157 (18)	0.60723 (19)	0.0230 (4)	
H2	0.9890	−0.2142	0.6630	0.028*	
C3	0.9665 (2)	−0.20684 (19)	0.5046 (2)	0.0275 (5)	
H3A	1.0123	−0.2557	0.4925	0.033*	
C4	0.9133 (2)	−0.15888 (19)	0.4223 (2)	0.0279 (5)	
H4	0.9241	−0.1735	0.3545	0.034*	
C5	0.8422 (2)	−0.08754 (18)	0.44056 (18)	0.0244 (5)	
C6	0.7806 (3)	−0.0350 (2)	0.3580 (2)	0.0334 (6)	
H6A	0.7886	−0.0474	0.2888	0.040*	
C7	0.7115 (3)	0.0315 (2)	0.3782 (2)	0.0340 (6)	
H7	0.6727	0.0641	0.3228	0.041*	
C8	0.6966 (2)	0.05305 (18)	0.48356 (19)	0.0252 (5)	
C9	0.6240 (2)	0.12042 (18)	0.5079 (2)	0.0284 (5)	
H9A	0.5856	0.1565	0.4562	0.034*	
C10	0.6100 (2)	0.13266 (18)	0.6084 (2)	0.0262 (5)	
H10A	0.5612	0.1762	0.6252	0.031*	
C11	0.6701 (2)	0.07880 (17)	0.68493 (18)	0.0203 (4)	
H11	0.6581	0.0859	0.7518	0.024*	
C12	0.75638 (19)	0.00351 (16)	0.56704 (18)	0.0191 (4)	
C13	0.53694 (19)	−0.33303 (16)	0.69398 (17)	0.0166 (4)	
C14	0.4237 (2)	−0.32036 (17)	0.69359 (17)	0.0184 (4)	
H14	0.4158	−0.2488	0.7164	0.022*	
C15	0.32329 (19)	−0.41601 (18)	0.65875 (18)	0.0201 (4)	
C16	0.3310 (2)	−0.52422 (17)	0.62553 (18)	0.0210 (4)	
H16A	0.2631	−0.5878	0.6035	0.025*	
C17	0.4446 (2)	−0.53255 (17)	0.62684 (17)	0.0192 (4)	
C18	0.54786 (19)	−0.43994 (17)	0.65935 (17)	0.0181 (4)	
H18	0.6227	−0.4492	0.6580	0.022*	
C19	0.64844 (19)	−0.23132 (17)	0.72999 (17)	0.0174 (4)	
C20	1.25799 (19)	0.17446 (16)	0.94754 (17)	0.0160 (4)	
C21	1.27380 (19)	0.26101 (17)	0.90393 (17)	0.0183 (4)	
H21	1.2048	0.2859	0.8671	0.022*	
C22	1.3936 (2)	0.30966 (17)	0.91613 (18)	0.0216 (4)	
C23	1.4972 (2)	0.27179 (18)	0.96526 (19)	0.0239 (5)	
H23	1.5765	0.3030	0.9700	0.029*	
C24	1.4771 (2)	0.18532 (18)	1.00693 (18)	0.0215 (4)	
C25	1.36097 (19)	0.13684 (17)	1.00183 (17)	0.0184 (4)	
H25	1.3520	0.0807	1.0338	0.022*	
C26	1.12560 (19)	0.11572 (16)	0.92589 (18)	0.0155 (4)	
C27	0.92602 (18)	0.29805 (17)	1.07147 (17)	0.0167 (4)	
C28	0.85225 (18)	0.33263 (17)	0.98083 (18)	0.0175 (4)	
H28	0.7983	0.2805	0.9039	0.021*	
C29	0.86033 (19)	0.44536 (17)	1.00666 (18)	0.0189 (4)	
C30	0.9405 (2)	0.52645 (17)	1.11932 (19)	0.0204 (4)	
H30	0.9449	0.6021	1.1355	0.024*	
C31	1.01314 (19)	0.48924 (17)	1.20606 (18)	0.0200 (4)	
C32	1.00898 (19)	0.37735 (17)	1.18544 (18)	0.0188 (4)	
H32	1.0604	0.3557	1.2463	0.023*	
C33	0.92031 (18)	0.17556 (17)	1.04534 (17)	0.0161 (4)	

### Atomic displacement parameters (Å^2^)

**Table:**

	*U*^11^	*U*^22^	*U*^33^	*U*^12^	*U*^13^	*U*^23^
Dy1	0.01184 (6)	0.01290 (6)	0.01367 (6)	0.00395 (4)	0.00583 (4)	0.00544 (4)
O1	0.0192 (7)	0.0182 (7)	0.0302 (8)	0.0036 (6)	0.0139 (7)	0.0055 (6)
O2	0.0177 (7)	0.0146 (7)	0.0228 (7)	0.0037 (6)	0.0084 (6)	0.0061 (6)
O3	0.0317 (10)	0.0325 (10)	0.0843 (16)	0.0094 (8)	0.0328 (11)	0.0032 (10)
O4	0.0200 (8)	0.0350 (9)	0.0456 (10)	−0.0006 (7)	0.0161 (8)	0.0094 (8)
O5	0.0296 (9)	0.0190 (8)	0.0611 (12)	0.0026 (7)	0.0168 (9)	0.0167 (8)
O6	0.0358 (9)	0.0256 (8)	0.0245 (8)	0.0157 (7)	0.0124 (7)	0.0088 (7)
O7	0.0205 (7)	0.0210 (7)	0.0218 (7)	0.0085 (6)	0.0135 (6)	0.0117 (6)
O8	0.0151 (7)	0.0235 (7)	0.0176 (7)	0.0012 (6)	0.0053 (6)	0.0074 (6)
O9	0.0382 (10)	0.0351 (9)	0.0442 (10)	0.0120 (8)	0.0206 (8)	0.0261 (8)
O10	0.0319 (9)	0.0325 (9)	0.0376 (9)	−0.0037 (7)	0.0192 (8)	0.0137 (8)
O11	0.0291 (10)	0.0643 (13)	0.0799 (15)	0.0235 (9)	0.0360 (10)	0.0402 (12)
O12	0.0256 (8)	0.0304 (8)	0.0245 (8)	0.0127 (7)	0.0092 (7)	0.0088 (7)
O13	0.0174 (7)	0.0177 (7)	0.0162 (7)	0.0068 (6)	0.0066 (6)	0.0045 (6)
O14	0.0183 (7)	0.0230 (7)	0.0182 (7)	0.0109 (6)	0.0088 (6)	0.0085 (6)
O15	0.0382 (9)	0.0293 (9)	0.0217 (8)	0.0133 (7)	0.0039 (7)	0.0075 (7)
O16	0.0403 (9)	0.0223 (8)	0.0333 (9)	0.0152 (7)	0.0181 (8)	0.0140 (7)
O17	0.0392 (10)	0.0231 (9)	0.0305 (9)	0.0028 (7)	0.0149 (8)	−0.0042 (7)
O18	0.0345 (10)	0.0388 (10)	0.0200 (8)	0.0059 (8)	0.0005 (7)	0.0031 (7)
N1	0.0192 (8)	0.0160 (8)	0.0184 (8)	0.0026 (7)	0.0084 (7)	0.0057 (7)
N2	0.0145 (8)	0.0154 (8)	0.0168 (8)	0.0014 (7)	0.0037 (7)	0.0053 (7)
N3	0.0202 (9)	0.0280 (10)	0.0295 (10)	0.0046 (8)	0.0123 (8)	0.0062 (8)
N4	0.0210 (9)	0.0167 (9)	0.0221 (9)	0.0027 (7)	0.0015 (8)	0.0059 (7)
N5	0.0306 (11)	0.0232 (9)	0.0257 (10)	0.0001 (8)	0.0144 (8)	0.0088 (8)
N6	0.0174 (9)	0.0303 (10)	0.0314 (10)	0.0075 (8)	0.0118 (8)	0.0089 (9)
N7	0.0239 (9)	0.0215 (9)	0.0241 (10)	0.0103 (8)	0.0119 (8)	0.0103 (8)
N8	0.0242 (10)	0.0245 (10)	0.0209 (9)	0.0006 (8)	0.0112 (8)	0.0003 (8)
C1	0.0193 (10)	0.0136 (9)	0.0166 (10)	−0.0027 (8)	0.0051 (8)	0.0035 (8)
C2	0.0247 (11)	0.0211 (10)	0.0234 (11)	0.0067 (9)	0.0130 (9)	0.0059 (9)
C3	0.0296 (12)	0.0234 (11)	0.0281 (12)	0.0051 (9)	0.0175 (10)	0.0027 (9)
C4	0.0312 (12)	0.0268 (11)	0.0189 (11)	−0.0020 (10)	0.0148 (10)	0.0001 (9)
C5	0.0268 (11)	0.0189 (10)	0.0168 (10)	−0.0047 (9)	0.0082 (9)	0.0020 (8)
C6	0.0458 (15)	0.0292 (12)	0.0175 (11)	0.0011 (11)	0.0128 (11)	0.0074 (9)
C7	0.0452 (15)	0.0291 (12)	0.0204 (11)	0.0062 (11)	0.0088 (11)	0.0131 (10)
C8	0.0275 (11)	0.0185 (10)	0.0194 (10)	0.0012 (9)	0.0039 (9)	0.0075 (8)
C9	0.0309 (12)	0.0205 (11)	0.0255 (11)	0.0068 (9)	0.0035 (10)	0.0128 (9)
C10	0.0229 (11)	0.0187 (10)	0.0280 (12)	0.0073 (9)	0.0039 (9)	0.0076 (9)
C11	0.0167 (10)	0.0182 (10)	0.0189 (10)	0.0038 (8)	0.0039 (8)	0.0047 (8)
C12	0.0173 (10)	0.0144 (9)	0.0162 (10)	−0.0017 (8)	0.0031 (8)	0.0038 (8)
C13	0.0172 (10)	0.0166 (10)	0.0125 (9)	0.0030 (8)	0.0042 (8)	0.0061 (8)
C14	0.0203 (10)	0.0177 (10)	0.0158 (10)	0.0049 (8)	0.0076 (8)	0.0061 (8)
C15	0.0170 (10)	0.0248 (11)	0.0184 (10)	0.0056 (8)	0.0080 (8)	0.0089 (8)
C16	0.0178 (10)	0.0201 (10)	0.0185 (10)	−0.0009 (8)	0.0049 (8)	0.0078 (8)
C17	0.0204 (10)	0.0162 (10)	0.0155 (9)	0.0042 (8)	0.0037 (8)	0.0063 (8)
C18	0.0165 (10)	0.0206 (10)	0.0157 (9)	0.0052 (8)	0.0056 (8)	0.0082 (8)
C19	0.0167 (10)	0.0187 (10)	0.0147 (9)	0.0034 (8)	0.0058 (8)	0.0072 (8)
C20	0.0173 (10)	0.0155 (9)	0.0136 (9)	0.0032 (8)	0.0085 (8)	0.0019 (8)
C21	0.0194 (10)	0.0179 (10)	0.0164 (10)	0.0043 (8)	0.0088 (8)	0.0047 (8)
C22	0.0252 (11)	0.0187 (10)	0.0202 (10)	0.0018 (8)	0.0124 (9)	0.0067 (8)
C23	0.0186 (10)	0.0253 (11)	0.0256 (11)	0.0010 (9)	0.0130 (9)	0.0056 (9)
C24	0.0174 (10)	0.0248 (11)	0.0200 (10)	0.0059 (8)	0.0085 (8)	0.0051 (9)
C25	0.0191 (10)	0.0203 (10)	0.0163 (10)	0.0056 (8)	0.0096 (8)	0.0056 (8)
C26	0.0166 (10)	0.0117 (9)	0.0206 (10)	0.0053 (7)	0.0109 (8)	0.0054 (8)
C27	0.0130 (9)	0.0204 (10)	0.0182 (10)	0.0054 (8)	0.0091 (8)	0.0063 (8)
C28	0.0144 (9)	0.0192 (10)	0.0173 (10)	0.0038 (8)	0.0079 (8)	0.0041 (8)
C29	0.0185 (10)	0.0205 (10)	0.0201 (10)	0.0073 (8)	0.0105 (8)	0.0079 (8)
C30	0.0203 (10)	0.0176 (10)	0.0253 (11)	0.0057 (8)	0.0143 (9)	0.0052 (8)
C31	0.0174 (10)	0.0216 (10)	0.0170 (10)	0.0015 (8)	0.0094 (8)	0.0018 (8)
C32	0.0161 (10)	0.0232 (10)	0.0170 (10)	0.0052 (8)	0.0081 (8)	0.0075 (8)
C33	0.0117 (9)	0.0199 (10)	0.0179 (10)	0.0055 (8)	0.0082 (8)	0.0058 (8)

### Geometric parameters (Å, °)

**Table:**

Dy1—O14^i^	2.3257 (13)	C3—H3A	0.9300
Dy1—O7^i^	2.3288 (14)	C4—C5	1.400 (3)
Dy1—O13	2.3362 (13)	C4—H4	0.9300
Dy1—O1	2.3865 (14)	C5—C6	1.437 (3)
Dy1—O8	2.4339 (14)	C6—C7	1.345 (4)
Dy1—O2	2.4702 (14)	C6—H6A	0.9300
Dy1—N2	2.4891 (16)	C7—C8	1.430 (3)
Dy1—N1	2.5593 (17)	C7—H7	0.9300
Dy1—O7	2.7576 (14)	C8—C9	1.404 (3)
Dy1—C19	2.768 (2)	C8—C12	1.410 (3)
Dy1—C26	2.9258 (19)	C9—C10	1.370 (3)
O1—C19	1.259 (3)	C9—H9A	0.9300
O2—C19	1.260 (2)	C10—C11	1.394 (3)
O3—N3	1.222 (3)	C10—H10A	0.9300
O4—N3	1.219 (2)	C11—H11	0.9300
O5—N4	1.232 (2)	C13—C18	1.386 (3)
O6—N4	1.218 (3)	C13—C14	1.390 (3)
O7—C26	1.258 (2)	C13—C19	1.504 (3)
O7—Dy1^i^	2.3287 (14)	C14—C15	1.381 (3)
O8—C26	1.250 (2)	C14—H14	0.9300
O9—N5	1.226 (3)	C15—C16	1.384 (3)
O10—N5	1.226 (3)	C16—C17	1.376 (3)
O11—N6	1.223 (3)	C16—H16A	0.9300
O12—N6	1.222 (3)	C17—C18	1.383 (3)
O13—C33	1.258 (2)	C18—H18	0.9300
O14—C33	1.251 (2)	C20—C21	1.388 (3)
O14—Dy1^i^	2.3256 (13)	C20—C25	1.390 (3)
O15—N7	1.224 (2)	C20—C26	1.501 (3)
O16—N7	1.220 (2)	C21—C22	1.382 (3)
O17—N8	1.218 (3)	C21—H21	0.9300
O18—N8	1.220 (3)	C22—C23	1.383 (3)
N1—C2	1.328 (3)	C23—C24	1.379 (3)
N1—C1	1.364 (3)	C23—H23	0.9300
N2—C11	1.328 (3)	C24—C25	1.383 (3)
N2—C12	1.359 (3)	C25—H25	0.9300
N3—C15	1.472 (3)	C27—C32	1.392 (3)
N4—C17	1.466 (3)	C27—C28	1.393 (3)
N5—C22	1.472 (3)	C27—C33	1.510 (3)
N6—C24	1.470 (3)	C28—C29	1.381 (3)
N7—C29	1.473 (3)	C28—H28	0.9300
N8—C31	1.481 (3)	C29—C30	1.385 (3)
C1—C5	1.410 (3)	C30—C31	1.377 (3)
C1—C12	1.437 (3)	C30—H30	0.9300
C2—C3	1.398 (3)	C31—C32	1.385 (3)
C2—H2	0.9300	C32—H32	0.9300
C3—C4	1.365 (3)		
			
O14^i^—Dy1—O7^i^	75.04 (5)	C3—C4—C5	119.7 (2)
O14^i^—Dy1—O13	132.09 (5)	C3—C4—H4	120.1
O7^i^—Dy1—O13	75.54 (5)	C5—C4—H4	120.1
O14^i^—Dy1—O1	75.25 (5)	C4—C5—C1	117.5 (2)
O7^i^—Dy1—O1	84.47 (5)	C4—C5—C6	123.7 (2)
O13—Dy1—O1	137.40 (5)	C1—C5—C6	118.8 (2)
O14^i^—Dy1—O8	87.69 (5)	C7—C6—C5	121.7 (2)
O7^i^—Dy1—O8	123.10 (5)	C7—C6—H6A	119.1
O13—Dy1—O8	77.86 (5)	C5—C6—H6A	119.1
O1—Dy1—O8	142.85 (5)	C6—C7—C8	121.1 (2)
O14^i^—Dy1—O2	124.20 (5)	C6—C7—H7	119.5
O7^i^—Dy1—O2	78.34 (5)	C8—C7—H7	119.5
O13—Dy1—O2	84.98 (5)	C9—C8—C12	117.9 (2)
O1—Dy1—O2	53.97 (5)	C9—C8—C7	123.3 (2)
O8—Dy1—O2	146.92 (5)	C12—C8—C7	118.9 (2)
O14^i^—Dy1—N2	142.26 (5)	C10—C9—C8	119.5 (2)
O7^i^—Dy1—N2	142.06 (5)	C10—C9—H9A	120.2
O13—Dy1—N2	77.89 (5)	C8—C9—H9A	120.2
O1—Dy1—N2	97.54 (5)	C9—C10—C11	118.9 (2)
O8—Dy1—N2	76.05 (5)	C9—C10—H10A	120.6
O2—Dy1—N2	72.78 (5)	C11—C10—H10A	120.6
O14^i^—Dy1—N1	77.58 (5)	N2—C11—C10	123.4 (2)
O7^i^—Dy1—N1	148.90 (5)	N2—C11—H11	118.3
O13—Dy1—N1	135.04 (5)	C10—C11—H11	118.3
O1—Dy1—N1	74.49 (5)	N2—C12—C8	122.0 (2)
O8—Dy1—N1	69.67 (5)	N2—C12—C1	117.75 (18)
O2—Dy1—N1	105.85 (5)	C8—C12—C1	120.21 (19)
N2—Dy1—N1	64.90 (6)	C18—C13—C14	120.33 (18)
O14^i^—Dy1—O7	67.13 (4)	C18—C13—C19	119.01 (18)
O7^i^—Dy1—O7	73.78 (5)	C14—C13—C19	120.65 (18)
O13—Dy1—O7	68.80 (4)	C15—C14—C13	118.76 (19)
O1—Dy1—O7	140.13 (5)	C15—C14—H14	120.6
O8—Dy1—O7	49.78 (4)	C13—C14—H14	120.6
O2—Dy1—O7	145.50 (4)	C14—C15—C16	122.8 (2)
N2—Dy1—O7	120.20 (5)	C14—C15—N3	119.53 (19)
N1—Dy1—O7	108.53 (5)	C16—C15—N3	117.71 (18)
O14^i^—Dy1—C19	99.17 (5)	C17—C16—C15	116.38 (19)
O7^i^—Dy1—C19	78.59 (5)	C17—C16—H16A	121.8
O13—Dy1—C19	110.97 (5)	C15—C16—H16A	121.8
O1—Dy1—C19	27.00 (6)	C16—C17—C18	123.46 (19)
O8—Dy1—C19	158.31 (5)	C16—C17—N4	117.99 (18)
O2—Dy1—C19	27.08 (5)	C18—C17—N4	118.54 (19)
N2—Dy1—C19	86.28 (5)	C17—C18—C13	118.28 (19)
N1—Dy1—C19	91.60 (6)	C17—C18—H18	120.9
O7—Dy1—C19	151.49 (5)	C13—C18—H18	120.9
O14^i^—Dy1—C26	73.35 (5)	O1—C19—O2	122.14 (18)
O7^i^—Dy1—C26	99.12 (5)	O1—C19—C13	118.19 (18)
O13—Dy1—C26	75.02 (5)	O2—C19—C13	119.66 (18)
O1—Dy1—C26	146.20 (5)	O1—C19—Dy1	59.36 (10)
O8—Dy1—C26	24.86 (5)	O2—C19—Dy1	63.16 (10)
O2—Dy1—C26	159.78 (5)	C13—C19—Dy1	172.09 (13)
N2—Dy1—C26	99.60 (5)	C21—C20—C25	120.43 (18)
N1—Dy1—C26	86.78 (5)	C21—C20—C26	118.89 (18)
O7—Dy1—C26	25.35 (5)	C25—C20—C26	120.47 (18)
C19—Dy1—C26	172.52 (6)	C22—C21—C20	119.00 (19)
C19—O1—Dy1	93.64 (12)	C22—C21—H21	120.5
C19—O2—Dy1	89.76 (11)	C20—C21—H21	120.5
C26—O7—Dy1^i^	168.95 (13)	C21—C22—C23	122.4 (2)
C26—O7—Dy1	84.82 (11)	C21—C22—N5	118.0 (2)
Dy1^i^—O7—Dy1	106.22 (5)	C23—C22—N5	119.59 (19)
C26—O8—Dy1	100.17 (12)	C24—C23—C22	116.61 (19)
C33—O13—Dy1	135.20 (12)	C24—C23—H23	121.7
C33—O14—Dy1^i^	133.49 (13)	C22—C23—H23	121.7
C2—N1—C1	117.54 (18)	C23—C24—C25	123.4 (2)
C2—N1—Dy1	124.43 (14)	C23—C24—N6	118.17 (19)
C1—N1—Dy1	117.88 (13)	C25—C24—N6	118.37 (19)
C11—N2—C12	118.20 (18)	C24—C25—C20	118.00 (19)
C11—N2—Dy1	120.97 (13)	C24—C25—H25	121.0
C12—N2—Dy1	120.55 (13)	C20—C25—H25	121.0
O4—N3—O3	123.91 (19)	O8—C26—O7	123.07 (18)
O4—N3—C15	118.31 (19)	O8—C26—C20	117.14 (17)
O3—N3—C15	117.78 (18)	O7—C26—C20	119.71 (17)
O6—N4—O5	124.60 (19)	O8—C26—Dy1	54.96 (10)
O6—N4—C17	118.01 (17)	O7—C26—Dy1	69.83 (10)
O5—N4—C17	117.39 (19)	C20—C26—Dy1	161.56 (13)
O9—N5—O10	124.19 (19)	C32—C27—C28	119.90 (18)
O9—N5—C22	117.89 (18)	C32—C27—C33	119.84 (18)
O10—N5—C22	117.9 (2)	C28—C27—C33	120.22 (17)
O12—N6—O11	124.3 (2)	C29—C28—C27	119.12 (18)
O12—N6—C24	118.18 (17)	C29—C28—H28	120.4
O11—N6—C24	117.53 (19)	C27—C28—H28	120.4
O16—N7—O15	124.06 (18)	C28—C29—C30	122.62 (19)
O16—N7—C29	118.20 (17)	C28—C29—N7	119.01 (18)
O15—N7—C29	117.72 (17)	C30—C29—N7	118.31 (18)
O17—N8—O18	124.96 (19)	C31—C30—C29	116.52 (19)
O17—N8—C31	117.57 (19)	C31—C30—H30	121.7
O18—N8—C31	117.47 (18)	C29—C30—H30	121.7
N1—C1—C5	122.7 (2)	C30—C31—C32	123.41 (19)
N1—C1—C12	117.96 (18)	C30—C31—N8	117.68 (19)
C5—C1—C12	119.38 (19)	C32—C31—N8	118.90 (19)
N1—C2—C3	123.4 (2)	C31—C32—C27	118.40 (19)
N1—C2—H2	118.3	C31—C32—H32	120.8
C3—C2—H2	118.3	C27—C32—H32	120.8
C4—C3—C2	119.1 (2)	O14—C33—O13	126.95 (19)
C4—C3—H3A	120.4	O14—C33—C27	116.68 (17)
C2—C3—H3A	120.4	O13—C33—C27	116.36 (17)
			
O14^i^—Dy1—O1—C19	151.62 (13)	C5—C1—C12—C8	−0.6 (3)
O7^i^—Dy1—O1—C19	75.66 (12)	C18—C13—C14—C15	0.3 (3)
O13—Dy1—O1—C19	14.09 (15)	C19—C13—C14—C15	179.34 (18)
O8—Dy1—O1—C19	−143.01 (11)	C13—C14—C15—C16	0.8 (3)
O2—Dy1—O1—C19	−3.93 (11)	C13—C14—C15—N3	−179.69 (18)
N2—Dy1—O1—C19	−66.19 (12)	O4—N3—C15—C14	179.0 (2)
N1—Dy1—O1—C19	−127.46 (13)	O3—N3—C15—C14	−0.8 (3)
O7—Dy1—O1—C19	132.03 (11)	O4—N3—C15—C16	−1.6 (3)
C26—Dy1—O1—C19	173.81 (11)	O3—N3—C15—C16	178.6 (2)
O14^i^—Dy1—O2—C19	−25.03 (13)	C14—C15—C16—C17	−1.1 (3)
O7^i^—Dy1—O2—C19	−87.70 (11)	N3—C15—C16—C17	179.44 (18)
O13—Dy1—O2—C19	−163.95 (12)	C15—C16—C17—C18	0.2 (3)
O1—Dy1—O2—C19	3.92 (11)	C15—C16—C17—N4	179.58 (18)
O8—Dy1—O2—C19	137.48 (12)	O6—N4—C17—C16	153.44 (19)
N2—Dy1—O2—C19	117.20 (12)	O5—N4—C17—C16	−27.0 (3)
N1—Dy1—O2—C19	60.54 (12)	O6—N4—C17—C18	−27.1 (3)
O7—Dy1—O2—C19	−124.20 (11)	O5—N4—C17—C18	152.43 (19)
C26—Dy1—O2—C19	−172.44 (14)	C16—C17—C18—C13	0.9 (3)
O14^i^—Dy1—O7—C26	99.42 (11)	N4—C17—C18—C13	−178.48 (17)
O7^i^—Dy1—O7—C26	179.69 (13)	C14—C13—C18—C17	−1.2 (3)
O13—Dy1—O7—C26	−99.87 (11)	C19—C13—C18—C17	179.82 (17)
O1—Dy1—O7—C26	120.03 (11)	Dy1—O1—C19—O2	7.4 (2)
O8—Dy1—O7—C26	−7.99 (10)	Dy1—O1—C19—C13	−171.36 (15)
O2—Dy1—O7—C26	−142.97 (11)	Dy1—O2—C19—O1	−7.11 (19)
N2—Dy1—O7—C26	−38.96 (12)	Dy1—O2—C19—C13	171.61 (16)
N1—Dy1—O7—C26	32.23 (12)	C18—C13—C19—O1	2.3 (3)
C19—Dy1—O7—C26	164.97 (12)	C14—C13—C19—O1	−176.74 (18)
O14^i^—Dy1—O7—Dy1^i^	−80.26 (6)	C18—C13—C19—O2	−176.48 (18)
O7^i^—Dy1—O7—Dy1^i^	0.0	C14—C13—C19—O2	4.5 (3)
O13—Dy1—O7—Dy1^i^	80.44 (6)	O14^i^—Dy1—C19—O1	−27.75 (12)
O1—Dy1—O7—Dy1^i^	−59.66 (9)	O7^i^—Dy1—C19—O1	−100.33 (12)
O8—Dy1—O7—Dy1^i^	172.32 (8)	O13—Dy1—C19—O1	−169.84 (11)
O2—Dy1—O7—Dy1^i^	37.35 (10)	O8—Dy1—C19—O1	79.41 (19)
N2—Dy1—O7—Dy1^i^	141.36 (6)	O2—Dy1—C19—O1	173.01 (19)
N1—Dy1—O7—Dy1^i^	−147.46 (5)	N2—Dy1—C19—O1	114.64 (12)
C19—Dy1—O7—Dy1^i^	−14.72 (13)	N1—Dy1—C19—O1	49.92 (12)
C26—Dy1—O7—Dy1^i^	−179.69 (13)	O7—Dy1—C19—O1	−85.92 (15)
O14^i^—Dy1—O8—C26	−53.49 (12)	O14^i^—Dy1—C19—O2	159.24 (11)
O7^i^—Dy1—O8—C26	16.95 (14)	O7^i^—Dy1—C19—O2	86.66 (11)
O13—Dy1—O8—C26	80.52 (12)	O13—Dy1—C19—O2	17.15 (12)
O1—Dy1—O8—C26	−115.11 (13)	O1—Dy1—C19—O2	−173.01 (19)
O2—Dy1—O8—C26	140.91 (12)	O8—Dy1—C19—O2	−93.60 (18)
N2—Dy1—O8—C26	160.86 (13)	N2—Dy1—C19—O2	−58.36 (11)
N1—Dy1—O8—C26	−131.10 (13)	N1—Dy1—C19—O2	−123.08 (11)
O7—Dy1—O8—C26	8.14 (11)	O7—Dy1—C19—O2	101.07 (14)
C19—Dy1—O8—C26	−162.75 (14)	C25—C20—C21—C22	−0.5 (3)
O14^i^—Dy1—O13—C33	−8.6 (2)	C26—C20—C21—C22	−175.35 (18)
O7^i^—Dy1—O13—C33	45.06 (18)	C20—C21—C22—C23	3.1 (3)
O1—Dy1—O13—C33	109.74 (18)	C20—C21—C22—N5	−176.46 (18)
O8—Dy1—O13—C33	−84.17 (18)	O9—N5—C22—C21	2.3 (3)
O2—Dy1—O13—C33	124.29 (18)	O10—N5—C22—C21	−179.54 (19)
N2—Dy1—O13—C33	−162.27 (19)	O9—N5—C22—C23	−177.3 (2)
N1—Dy1—O13—C33	−128.25 (17)	O10—N5—C22—C23	0.9 (3)
O7—Dy1—O13—C33	−32.85 (17)	C21—C22—C23—C24	−2.8 (3)
C19—Dy1—O13—C33	116.54 (18)	N5—C22—C23—C24	176.79 (19)
C26—Dy1—O13—C33	−58.74 (18)	C22—C23—C24—C25	−0.2 (3)
O14^i^—Dy1—N1—C2	7.15 (15)	C22—C23—C24—N6	178.21 (19)
O7^i^—Dy1—N1—C2	−21.6 (2)	O12—N6—C24—C23	162.5 (2)
O13—Dy1—N1—C2	145.80 (14)	O11—N6—C24—C23	−19.1 (3)
O1—Dy1—N1—C2	−70.76 (16)	O12—N6—C24—C25	−19.1 (3)
O8—Dy1—N1—C2	99.30 (16)	O11—N6—C24—C25	159.4 (2)
O2—Dy1—N1—C2	−115.25 (16)	C23—C24—C25—C20	2.6 (3)
N2—Dy1—N1—C2	−177.03 (17)	N6—C24—C25—C20	−175.74 (18)
O7—Dy1—N1—C2	67.58 (16)	C21—C20—C25—C24	−2.3 (3)
C19—Dy1—N1—C2	−91.89 (16)	C26—C20—C25—C24	172.53 (18)
C26—Dy1—N1—C2	80.80 (16)	Dy1—O8—C26—O7	−16.4 (2)
O14^i^—Dy1—N1—C1	−168.30 (14)	Dy1—O8—C26—C20	160.48 (14)
O7^i^—Dy1—N1—C1	162.97 (12)	Dy1^i^—O7—C26—O8	−167.3 (5)
O13—Dy1—N1—C1	−29.65 (17)	Dy1—O7—C26—O8	14.28 (18)
O1—Dy1—N1—C1	113.79 (14)	Dy1^i^—O7—C26—C20	15.9 (8)
O8—Dy1—N1—C1	−76.15 (14)	Dy1—O7—C26—C20	−162.55 (16)
O2—Dy1—N1—C1	69.30 (14)	Dy1^i^—O7—C26—Dy1	178.4 (7)
N2—Dy1—N1—C1	7.52 (13)	C21—C20—C26—O8	40.1 (3)
O7—Dy1—N1—C1	−107.87 (13)	C25—C20—C26—O8	−134.8 (2)
C19—Dy1—N1—C1	92.66 (14)	C21—C20—C26—O7	−142.91 (19)
C26—Dy1—N1—C1	−94.65 (14)	C25—C20—C26—O7	42.2 (3)
O14^i^—Dy1—N2—C11	−175.70 (13)	C21—C20—C26—Dy1	99.9 (4)
O7^i^—Dy1—N2—C11	18.05 (19)	C25—C20—C26—Dy1	−74.9 (5)
O13—Dy1—N2—C11	−28.27 (14)	O14^i^—Dy1—C26—O8	123.04 (12)
O1—Dy1—N2—C11	108.70 (15)	O7^i^—Dy1—C26—O8	−165.68 (12)
O8—Dy1—N2—C11	−108.59 (15)	O13—Dy1—C26—O8	−93.42 (12)
O2—Dy1—N2—C11	60.17 (15)	O1—Dy1—C26—O8	100.63 (14)
N1—Dy1—N2—C11	177.62 (16)	O2—Dy1—C26—O8	−84.67 (19)
O7—Dy1—N2—C11	−84.71 (15)	N2—Dy1—C26—O8	−18.82 (13)
C19—Dy1—N2—C11	84.11 (15)	N1—Dy1—C26—O8	45.05 (12)
C26—Dy1—N2—C11	−100.55 (15)	O7—Dy1—C26—O8	−165.38 (19)
O14^i^—Dy1—N2—C12	−1.93 (18)	O14^i^—Dy1—C26—O7	−71.58 (11)
O7^i^—Dy1—N2—C12	−168.19 (12)	O7^i^—Dy1—C26—O7	−0.30 (13)
O13—Dy1—N2—C12	145.49 (14)	O13—Dy1—C26—O7	71.95 (11)
O1—Dy1—N2—C12	−77.54 (14)	O1—Dy1—C26—O7	−93.99 (13)
O8—Dy1—N2—C12	65.18 (14)	O8—Dy1—C26—O7	165.38 (19)
O2—Dy1—N2—C12	−126.07 (14)	O2—Dy1—C26—O7	80.71 (18)
N1—Dy1—N2—C12	−8.62 (13)	N2—Dy1—C26—O7	146.55 (11)
O7—Dy1—N2—C12	89.06 (14)	N1—Dy1—C26—O7	−149.57 (11)
C19—Dy1—N2—C12	−102.13 (14)	O14^i^—Dy1—C26—C20	53.0 (4)
C26—Dy1—N2—C12	73.21 (14)	O7^i^—Dy1—C26—C20	124.3 (4)
C2—N1—C1—C5	−1.3 (3)	O13—Dy1—C26—C20	−163.5 (4)
Dy1—N1—C1—C5	174.49 (14)	O1—Dy1—C26—C20	30.6 (5)
C2—N1—C1—C12	177.97 (18)	O8—Dy1—C26—C20	−70.0 (4)
Dy1—N1—C1—C12	−6.3 (2)	O2—Dy1—C26—C20	−154.7 (4)
C1—N1—C2—C3	0.7 (3)	N2—Dy1—C26—C20	−88.9 (4)
Dy1—N1—C2—C3	−174.77 (15)	N1—Dy1—C26—C20	−25.0 (4)
N1—C2—C3—C4	0.7 (3)	O7—Dy1—C26—C20	124.6 (5)
C2—C3—C4—C5	−1.5 (3)	C32—C27—C28—C29	−1.7 (3)
C3—C4—C5—C1	0.9 (3)	C33—C27—C28—C29	−179.22 (18)
C3—C4—C5—C6	−178.8 (2)	C27—C28—C29—C30	0.9 (3)
N1—C1—C5—C4	0.5 (3)	C27—C28—C29—N7	178.08 (18)
C12—C1—C5—C4	−178.73 (18)	O16—N7—C29—C28	−178.27 (19)
N1—C1—C5—C6	−179.83 (19)	O15—N7—C29—C28	0.1 (3)
C12—C1—C5—C6	0.9 (3)	O16—N7—C29—C30	−0.9 (3)
C4—C5—C6—C7	178.9 (2)	O15—N7—C29—C30	177.42 (19)
C1—C5—C6—C7	−0.7 (3)	C28—C29—C30—C31	0.0 (3)
C5—C6—C7—C8	0.1 (4)	N7—C29—C30—C31	−177.21 (18)
C6—C7—C8—C9	−179.0 (2)	C29—C30—C31—C32	−0.1 (3)
C6—C7—C8—C12	0.2 (3)	C29—C30—C31—N8	179.07 (18)
C12—C8—C9—C10	−2.2 (3)	O17—N8—C31—C30	8.0 (3)
C7—C8—C9—C10	177.1 (2)	O18—N8—C31—C30	−172.3 (2)
C8—C9—C10—C11	0.9 (3)	O17—N8—C31—C32	−172.78 (19)
C12—N2—C11—C10	−2.7 (3)	O18—N8—C31—C32	6.9 (3)
Dy1—N2—C11—C10	171.18 (15)	C30—C31—C32—C27	−0.7 (3)
C9—C10—C11—N2	1.7 (3)	N8—C31—C32—C27	−179.90 (18)
C11—N2—C12—C8	1.3 (3)	C28—C27—C32—C31	1.6 (3)
Dy1—N2—C12—C8	−172.66 (14)	C33—C27—C32—C31	179.17 (18)
C11—N2—C12—C1	−176.93 (17)	Dy1^i^—O14—C33—O13	50.0 (3)
Dy1—N2—C12—C1	9.1 (2)	Dy1^i^—O14—C33—C27	−128.83 (16)
C9—C8—C12—N2	1.1 (3)	Dy1—O13—C33—O14	−27.4 (3)
C7—C8—C12—N2	−178.17 (19)	Dy1—O13—C33—C27	151.46 (13)
C9—C8—C12—C1	179.28 (18)	C32—C27—C33—O14	9.9 (3)
C7—C8—C12—C1	0.0 (3)	C28—C27—C33—O14	−172.54 (18)
N1—C1—C12—N2	−1.6 (3)	C32—C27—C33—O13	−169.07 (18)
C5—C1—C12—N2	177.65 (17)	C28—C27—C33—O13	8.5 (3)
N1—C1—C12—C8	−179.85 (18)		

Symmetry codes: (i) −*x*+2, −*y*, −*z*+2.
